# IFN-Aging: Coupling Aging With Interferon Response

**DOI:** 10.3389/fragi.2022.870489

**Published:** 2022-05-02

**Authors:** Wei Cao

**Affiliations:** Department of Anesthesiology, McGovern Medical School, University of Texas Health Science Center at Houston, Houston, TX, United States

**Keywords:** aging, interferon, senescence, inflammaging, transposable elements, laminopathy, mitochondria

## Abstract

Chronic inflammation affects many diseases and conditions, including aging. Interferons are a part of the immune defense against viral infections. Paradoxically, various aging tissues and organs from mammalian hosts perpetually accumulate changes brought by interferon pathway activation. Herein, we connote the mechanisms behind this phenomenon and discuss its implications in age-related pathology.

## Introduction

Chronic inflammation is associated with many known pathologies, ranging from cardiovascular disease to cancer. Mounting evidence now links chronic inflammation to aging, a phenomenon commonly referred to as inflammaging (Franceschi and Campisi 2014; Kennedy et al., 2014). Evolutionarily, inflammation is a cellular mechanism to clear invading pathogens or damaged tissue and coordinate repair and restoration. The protective inflammatory response is transient and tightly controlled by the coordinated actions from copious pro- and anti-inflammatory factors.

Although early studies revealed that aging mammalian hosts generally display increased systemic inflammation, the degree and nature of tissue- and organ-level inflammation remained unclear. Recently, profound insights on the core signature related to tissue aging arose from several large-scale unbiased surveys of key tissues, organs, and cells from different hosts at various intervals during their lifespan.

### Interferon Activation Accompanies All Tissue Aging

Benayoun et al. collected heart, liver, olfactory bulb, and primary neural stem cells from mice at three different ages and simultaneously analyzed their epigenomes and transcriptomes (Benayoun et al., 2019). This comprehensive study identified recurrent age-related chromatin and transcriptional changes in the upregulation of immune system response pathways, particularly the interferon (IFN) response, in all mouse tissues. Intriguingly, the 16 genes upregulated with age across tissues are responsive to interferon, and the phenomenon of age-related innate immune activation is seemingly applicable to humans. Shavlakadze et al. compared liver, skeletal muscle, kidney, and the hippocampus at seven different age points of rats and, *via* gene expression profiling, discovered a progressive upregulation of innate immune responses, including IFN responses, in the tissues (Shavlakadze et al., 2019). Among the 11 annotated genes jointly upregulated in all four tissues, the majority are inducible by interferon. Angelidis et al. conducted concomitant transcriptome and proteome analysis and revealed a similar upregulation of immune activation, including the interferon response, focusing on cells isolated from young *versus* old mouse lungs (Angelidis et al., 2019). Moreover, Baruch et al. compared multiple aged tissues and identified the choroid plexus as a locus within the aging brain that expresses extraordinarily high IFN signature (Baruch et al., 2014). Despite pending confirmation in humans, these studies collectively highlight that progressive inflammation, in particular IFN response, constitutes a conserved core process related to tissue and organ aging, a term coined here as IFN-aging.

### Forces Driving Age-Related Interferon Response

From these intriguing discoveries and other recent research development, a paradigm that blends the fundamental principles of molecular biology and immunology starts to emerge. The mammalian genome encodes three types of interferons–types I (primarily IFNα/β), II (IFNγ), and III (IFNλ), among which types I (IFN-I) and III IFN are induced *via* innate immune responses upon sensing of microbial nucleic acids by innate immune receptors, such as cGAS (Roers et al., 2016). In the absence of pathogen infection, host-derived nucleic acids may activate the IFN pathway aberrantly, a process now postulated to take place during aging.

Constituting >40% of the human and rodent genomes, transposable elements (TE), with viral reminiscence, are routinely repressed in somatic cells to prevent their mobilization (Bourque et al., 2018; Makałowski et al., 2019). The “transposon theory of aging” posits that the cellular control mechanism that keeps TEs in check declines with age and contributes to age-related tissue dysfunction (Murray 1990; De Cecco et al., 2013; Li et al., 2013; Gorbunova et al., 2014; Wood et al., 2016; Gorbunova et al., 2021). *In vitro*, primary cells under replicative or other stress can enter a state of growth arrest named senescence. While undergoing other cellular and molecular changes, these cells secrete proinflammatory factors, a feature termed senescence-associated secretive phenotype (SASP) (Coppé et al., 2008). At a late stage, senescent cells accumulate cytosolic DNA from activated LINE-1, a family of TEs, which activates the cGAS-STING innate signaling pathway to initiate IFN-I response as part of full SASP (Franceschi and Campisi 2014; De Cecco et al., 2019). TE derepression and cGAS-STING-IFN innate signaling is predominant in a group of diseases called laminopathy, including several conditions of accelerated aging (Graziano et al., 2018). Mechanical stress from defective nuclear lamina can disrupt the heterochromatin structure that normally silences LINE-1, which leads to its activation and subsequent immune response (Kreienkamp et al., 2018; Earle et al., 2020). Sirtuins are a class of nicotinamide adenine dinucleotide-consuming enzymes implicated in diverse biological pathways. In particular, SIRT3 and SIRT6 critically stabilize the nuclear lamina and maintain TE quiescence as the cells or animals lacking these genes undergo senescence or premature aging, respectively (Simon et al., 2019; Diao et al., 2021).

Genomic instability and epigenetic alterations are key hallmarks of aging (López-Otín et al., 2013). DNA damage affects many aspects of the aging phenotype, and genetic mutations affecting genome maintenance and repair promote the premature onset of age-related pathologies (Rieckher et al., 2021; Schumacher et al., 2021). Double-stranded DNA damage is sufficient to induce IFNβ production, which stimulates the p53 pathway and promotes senescence (Yu et al., 2015). While age-associated epigenetic alterations weaken the genome surveillance mechanism and increase TE transposition, manipulating genes known to affect heterochromatin structure mitigate the expression of TEs and extend life span (Wood et al., 2016). Not coincidentally, laminopathy often manifests with DNA damage, epigenome alteration, and loss of genomic integrity (Shumaker et al., 2006; Kreienkamp et al., 2018; Earle et al., 2020). In keeping with these mechanisms, Benayoun et al. detected TE activation in conjunction with epigenetic reprogramming in the aging tissues, a finding consistent with the profound upturn of IFN response (Benayoun et al., 2019).

**FIGURE 1:**
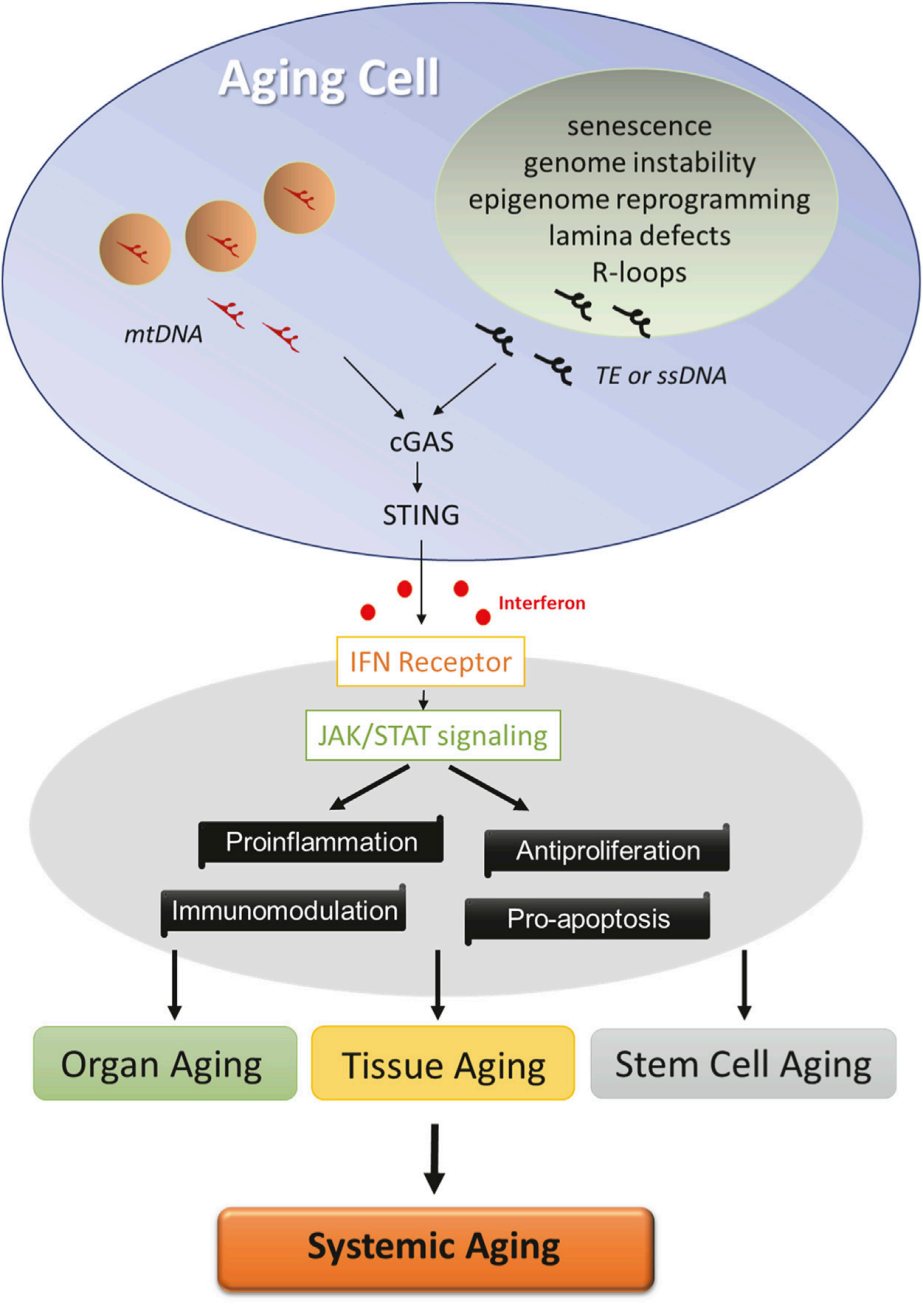
Factors driving age-related IFN response. Cells undergo multiple molecular changes in the nuclei (green) and mitochondria (pink) during aging. Cytosolic sensing of TE, ssDNA, or mitochondrial DNA by cGAS activates STING and initiates innate interferon response in aging cells. Interferon signaling *via* JAK/STAT pathway exerts a profound influence on cellular functions, and its activation constitutes a core program of tissue, organ, and stem cell aging, which likely contributes to the systemic aging process.

Besides nuclear defects, aging cells accumulate mutations and deletions in the mitochondrial genome. The “mitochondrial theory of aging” denotes that accumulation of damage to mitochondria and mitochondria DNA (mtDNA) leads to aging of humans and animals, in line with the accelerated aging of mtDNA mutator mice (Trifunovic et al., 2004; Kujoth et al., 2005; Jang et al., 2018). mtDNA evolutionarily resembles bacterial DNA, an agonist for innate IFN response. mtDNA stress stimulates the escape of mtDNA into the cytosol, where it engages with the cGAS-STING pathway to initiate IFN response (West et al., 2015). Intriguingly, genetic ablation of the IFN-I receptor not only attenuates the hyperinflammatory phenotypes but also extends the life span of the mtDNA mutator mice (Lei et al., 2021). Altogether, these studies reveal a causal relationship between age-associated nuclear or mitochondrial dysregulation and innate IFN response (Figure 1).

## Concluding Remarks

The recent discovery of IFN-aging has important implications not only in basic research but also in translational perspectives. Nucleoside reverse-transcriptase inhibitors (NRTi) are a class of FDA-approved drugs that suppress TE by interfering with the reverse transcription step of retroelement replication (Jones et al., 2008; Dai et al., 2011). Treatment with NRTi effectively decreased TE transposition, dampened IFN response, and extended the life span of aging mice and *Drosophila* (Wood et al., 2016; Simon et al., 2019), consistent with the driver role of TE derepression in aging. Separately, calorie restriction extends the life span of *Drosophila* in conjunction with decreased TE transposition (Wood et al., 2016). One of the exciting developments of the aging field is the success of senolytic compounds in eliminating senescent cells from tissues and rejuvenating the aging host *in vivo* (Baker et al., 2011; Xu et al., 2018), albeit its specific impact on tissue IFN response remains to be established.

The conceptual breakthrough of the IFN-aging phenomenon undoubtedly invites studies to illuminate several key questions to shed more light on the core aging process:

1) What are the cell types in each tissue or organ most vulnerable to IFN activation? Are they all senescent cells? Besides stromal and other cell types, tissue macrophages can accumulate age-dependent DNA damage and polarize towards a proinflammatory state (Horn and Triantafyllopoulou 2018; Guimarães et al., 2021). With technical improvement, omic approaches should help generate a refined tissue aging map at a single-cell level in the future (He et al., 2020).
2) In addition to TEs and mtDNA, aberrant R-loop structures containing RNA/DNA hybrids are linked to chronic inflammation and human diseases (Richard and Manley 2017). Interestingly, R-loops in aged pancreatic cells trigger the release of cytoplasmic single-stranded DNA (ssDNA), leading to IFN activation in a STING-dependent manner (Chatzidoukaki et al., 2021). How central is the cGAS-STING axis in tissue versus organismic aging? Do other nucleic acid-sensing immune pathways participate in IFN-aging?
3) With aging, white adipose tissue cells are highly susceptible to DNA damage and senescence (Smith et al., 2021). These adipocytes elicit a chronic autoinflammatory response, which is significantly amplified by obesity-driven IFN activation (Karakasilioti et al., 2013; Chan et al., 2020). How does IFN contribute to the aging of specific tissue? Is there any functional divergence among the IFN subtypes? This is an important point to clarify as IFN can exert proinflammatory, antiproliferative, proapoptotic, and immunomodulatory functions in a contact-dependent manner (Ng et al., 2016; Burke and Young 2019).
4) Aging of the immune system impacts the morbidity and mortality of the elderly. DNA damage in bone marrow stem cells can induce IFNβ production, which leads to immune stem cell senescence and decline (Yu et al., 2015). Strikingly, the senescent immune cells are sufficient to trigger systemic aging, as non-lymphoid organs also show increased senescence and damage in mice, despite restricted DNA damage in hematopoietic cells (Yousefzadeh et al., 2021). How important is IFN in organismic aging is yet to be fully elucidated.

Answers to all these questions would greatly facilitate the eventual development of targeted therapeutics to modify age-related processes and pathologies.
